# The daily swab test collection problem

**DOI:** 10.1007/s10479-022-05019-1

**Published:** 2022-10-27

**Authors:** Roberto Aringhieri, Sara Bigharaz, Alessandro Druetto, Davide Duma, Andrea Grosso, Alberto Guastalla

**Affiliations:** 1 Dipartimento di Informatica, Università degli Studi di Torino, Corso Svizzera 185, 10149 Torino, Italy; 2 Department of Industrial Economics and Technology Management, Faculty of Economics and Management, NTNU, 7491 Trondheim, Norway; 3 Dipartimento di Matematica “Felice Casorati”, Università degli Studi di Pavia, via Adolfo Ferrata, 5, 27100 Pavia, Italy

**Keywords:** OR in health services, Covid-19, Digital contact tracing, Team orienteering problem, Hybrid algorithms

## Abstract

Digital Contact Tracing (DCT) has been proved to be an effective tool to counteract the new SARS-CoV-2 or Covid-19. Despite this widespread effort to adopt the DCT, less attention has been paid to the organisation of the health logistics system that should support the tracing activities. Actually, the DCT poses a challenge to the logistics of the local health system in terms of number of daily tests to be collected and evaluated, especially when the spreading of the virus is soaring. In this paper we introduce a new optimisation problem called the Daily Swab Test Collection (DSTC) problem, that is the daily problem of collecting swab tests at home in such a way to guarantee a timely testing to people notified by the app to be in contact with a positive case. The problem is formulated as a variant of the team orienteering problem. The contributions of this paper are the following: (i) the new optimisation problem DSTC that complements and improves the DCT approach proposed by Ferretti et al. (Science 10.1126/science.abb6936, 2020), (ii) the DSCT formulation as a variant of the TOP and a literature review highlighting that this variant can have useful application in healthcare management, (iii) new realistic benchmark instances for the DSTC based on the city of Turin, (iv) two new efficient and effective hybrid algorithms capable to deal with realistic instances, (v) the managerial insights of our approach with a special regard on the fairness of the solutions. The main finding is that it possible to optimise the underlying logistics system in such a way to guarantee a timely testing to people recognised by the DCT.

## Introduction

Contact tracing is one of the key tools to avoid or to limit an outbreak of a pandemic disease. Unfortunately, manual contact tracing could be not efficient as soon as the speed of spread disease increases. Digital contact tracing (DCT) has been experimentally proved to be an effective tool to counteract the new SARS-CoV-2 or Covid-19, as discussed in Ferretti et al. (2020). The basic idea is to implement the DCT through a smartphone app which builds a list of proximity contacts and immediately notifies contacts of positive cases. The authors proved that viral spread is too fast to be contained by manual contact tracing, but could be controlled if this process was faster, more efficient and implemented at a large-scale. In accordance with their research findings, their models show that it is possible to stop the epidemic if the adoption rate of the DCT app is approximately 60% of the population, even if the DCT has an effect at all levels of uptake.

Many countries in the world launched its own DCT app. Despite this widespread effort, less attention has been posed to the organisation of the health logistic system that should support the tracing activities. To the best of our knowledge, this is the first paper dealing with the healthcare logistics underlying a DCT system from an Operations Research perspective. Accordingly, the DCT poses a challenge to the logistic of the local health system in terms of number of daily tests to be collected and evaluated, especially when the spreading of the virus is soaring. For instance, the number of daily tests in Italy increases from 250, 000 on average in September 2021 up to 1, 000, 000 on average in January 2022 (lab24.ilsole24ore.com/coronavirus/). In that period the DCT was available in Italy and many newspaper reported really long queues and the inability to access the testing service for the elderly.

As a matter of fact, the success of the DCT is directly related to the ability of the local health system to test the majority of the contacts of a positive case as soon as possible. By consequence the following research question arises: is it possible to optimise the underlying logistics system in such a way to guarantee a timely testing to people notified by the app to be in contact with a positive case?

In this paper we introduce a new optimisation problem called the Daily Swab Test Collection (DSTC) problem, that is the daily problem of collecting swab tests at home of those people likely to be positive in accordance with the guidelines described by Ferretti et al. (2020). The DSTC can be formulated as a variant of the Team Orienteering Problem (TOP) (Butt and Cavalier, 1994; Chao et al., 1996b). We generate a new set of realistic benchmark instances based on the city of Turin. We propose two new hybrid algorithms based on machine learning and a neighbourhood search, which are capable to largely reduce the solution running time with respect to those computed by a general purpose solver, especially when the complexity of the problem increases.

In summary, the contributions of this study are the following: (i) the new optimisation problem DSTC that complements and improves the DCT approach proposed by Ferretti et al. (2020), (ii) the DSCT formulation as a variant of the TOP and a literature review highlighting that this variant can have useful application in healthcare management, (iii) new realistic benchmark instances for the DSTC based on the city of Turin, (iv) two new efficient and effective hybrid algorithms capable to deal with realistic instances, (v) the managerial insights of our approach with a special regard on the fairness of the solutions.

The paper is organised as follows. After a more detailed description of the DCT and a literature review in Sect. 2, the problem statement and an integer linear programming model for the DSTC are reported in Sect. 3. The solution algorithms are described in Sect. 4. The quantitative analysis based on the set of the new realistic instances is reported and discussed in Sect. 5. Conclusions, challenges and future works are discussed in Sect. 6.

## Literature review

**Fig. 1:**
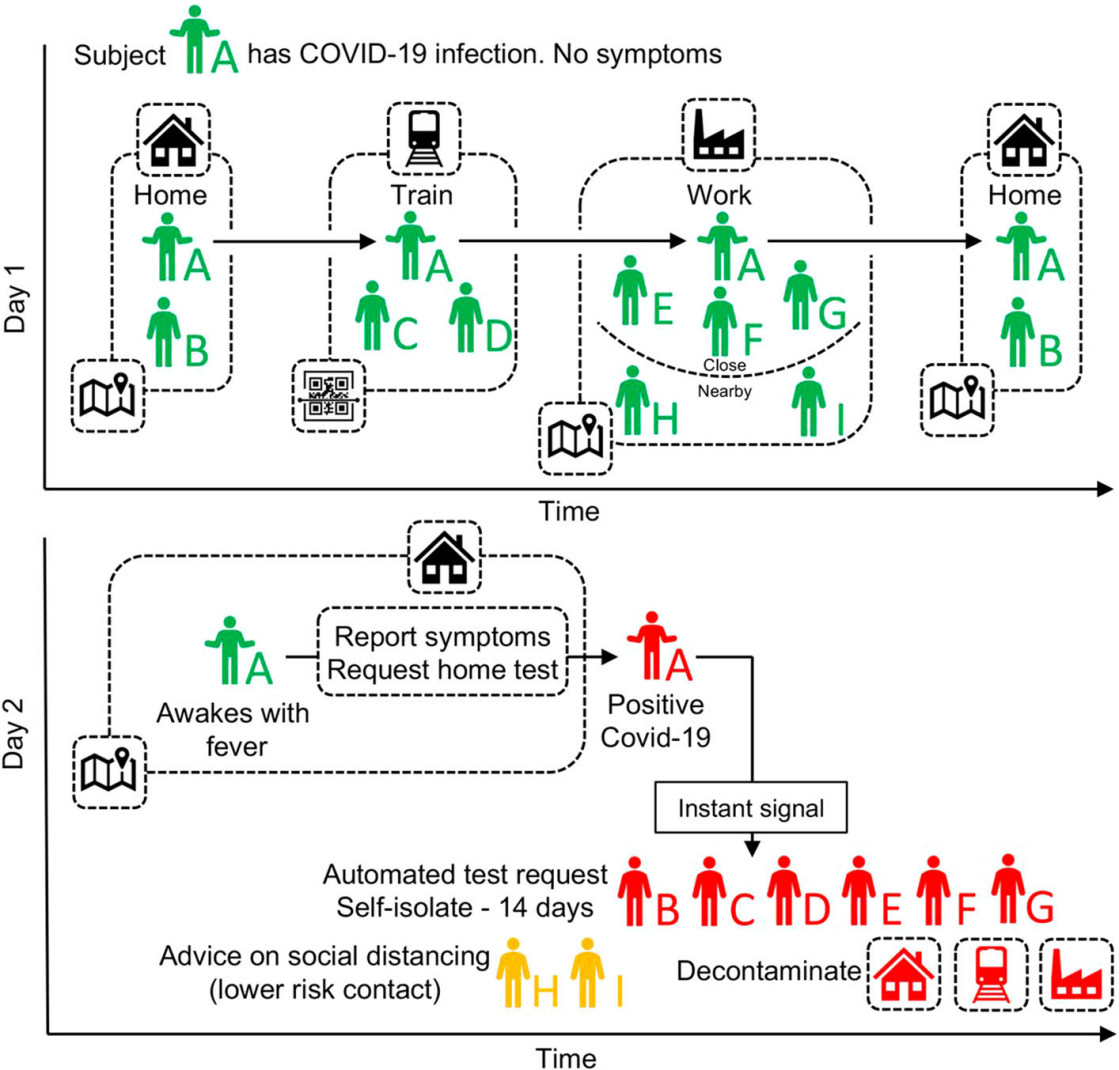
General description of digital contact tracing (from Ferretti et al. (2020))

The DCT can be a fundamental component of the *triple T strategy*, that is *test*, *trace* and *treat*. Basically, it consists of a smartphone app for automatically tracing the contacts of people in order to trace them faster as soon as a new case occurs as proposed by Ferretti et al. (2020) and depicted in Fig. 1: after reporting the main symptoms (e.g., fever, cough, ...), the potential new case starts isolation and requests home test. Such a test should be collected and evaluated as soon as possible in order to alert all the people in contact with her/him. But if the test is delayed, the benefits of the DCT can be wasted.

Many countries in the world launched its own DCT app, especially in Europe so much that the EU commission has set up an EU-wide system to ensure interoperability among the DCT apps in order to exploit their full potential to break the chain of Covid-19 infections across borders and save lives^1^.

Wymant et al. (2021) investigated the impact of the NHS COVID-19 app for England and Wales, from its launch on 24 September 2020 through to the end of December 2020. It was used regularly by approximately the $28\%$ of the total population sending approximately 1.7 million exposure notifications resulting in about the $6.0\%$ of individuals subsequently showing symptoms and testing positive. The authors estimated that for every percentage point increase in app users, the number of cases can be reduced by $0.8\%$ (modelling) or $2.3\%$ (statistical analysis). These findings provide evidence for continued development and deployment of such apps.

As reported in Fig. 1, the closest contacts of a positive case require a swab test as soon as possible. Such a swab test should be done at home due to self-isolation. Further, the number of swab tests can become larger and larger: the number of daily tests in the province of Turin increases from 50, 000 on average in September 2021 up to 100, 000 on average in January 2022 (lab24.ilsole24ore.com/coronavirus/) determining long waiting queues and limiting the access for the elderly to the testing service.

Therefore the need of a quantitative model-like the DSTC-to support the inherent health logistics of the DCT is evident. Basically the DSTC consists in organising the daily collection of swab tests reaching the house of the contact(s) of a positive case detected the day(s) before. A set of teams are in charge of collecting the swab tests around the city.

The DSTC could be modelled as a variant of the TOP (see, e.g., Vansteenwegen et al. (2011); Gunawan et al. (2016); Vansteenwegen and Gunawan (2019) for more detailed literature reviews). The TOP is a routing problem belonging to the class of the Vehicle Routing Problems with Profits, which are characterised by the fact that not all customers can be served unlike the classical Vehicle Routing Problem. This implies the need to consider two different decisions (Archetti et al. 2014), that is (i) which customers to serve, and (ii) how to cluster the customers to be served in different routes (if more than one) and order the visits in each route. The customer selection is driven by a profit associated with each customer that makes such a customer more or less attractive.

The DSTC differs from the TOP for the fact that a non-negligible service time at the customer should be considered. Erdogan and Laporte (2013) introduces the Orienteering Problem with Variable Profits (OPVP) in which a single vehicle can collect the whole profit at the customer after a discrete number of “passes” or spending a continuous amount of time. As in the classical orienteering problem, the objective is to determine a maximal profit tour for the vehicle, starting and ending at the depot, and not exceeding a travel time limit. A well-known application of the OPVP is the Tourist Trip Design Problem (TTDP): in the TTDP, the main challenges are (i) the decision of which Points Of Interest (POIs) should be visited, and (ii) to determine the best sequence for the trip day. In accordance with Vansteenwegen and Gunawan (2019) (chapter 7), the multiple day TTDP can be formulated as the TOP in which days are modelled as teams.

The aim of the work of Gavalas et al. (2014) is to survey models, algorithmic approaches and methodologies concerning the TTDP. Literature approaches were examined, focusing on problem models that best capture a multitude of realistic POIs attributes and user constraints. In Yu et al. (2019), the authors consider the case in which a duration of the attraction visit is considered, and the score of visiting an attraction is different depending on the time of visit. The authors proposed a hybrid artificial bee colony optimisation algorithm to solve instances with 25, 50, and 100 locations and up to 4 tours. Exposito et al. (2019) consider an extension of the TTDP, named TTDP with Clustered POIs, where they are grouped in clusters representing different types of attraction sites. The authors proposed a Fuzzy Greedy Randomized Adaptive Search Procedure for solving this problem, in which both distance based and score based evaluation criteria are used to guide the candidates selection in the construction phase. More recently, the study in Moosavi Heris et al. (2022) aims (i) to maximise the profit from visiting the POIs to increase the satisfaction level, and (ii) to maximise the number of visited POIs to improve the satisfaction level of the tourists. The authors introduced the concepts of “indirect coverage” and “neighbourhood radius” to increase the accessibility of the tourist attractions and POIs. Solutions of the problem are computed using CPLEX (for small-scale instances) and NSGA-II algorithm (for large-scale instances).

Few contributions are available in the literature outside the TTDP operative context. Lin and Yu (2017) investigates a new variant of the TOP with time windows in which some customers are important customers that must be visited. The other customers are called optional customers. Each customer carries a positive score and service time. The goal is to determine a given number of paths to maximise the total score collected at visited nodes, while observing side constraints such as mandatory visits and time window constraints. The authors proposed a multi-start simulated annealing (MSA) heuristic for this problem. Stavropoulou et al. (2019) introduces the Consistent Vehicle Routing Problem with Profits as a variant of the TOP. In this problem there are two sets of customers, the frequent customers that are mandatory to service and the non-frequent potential customers with known and estimated profits respectively, both having known demands and service requirements over a planning horizon of multiple days. The objective is to determine the vehicle routes that maximise the net profit, while satisfying vehicle capacity, route duration and consistency constraints. For addressing this computationally challenging problem, an Adaptive Tabu Search has been developed, utilising both short- and long-term memory structures to guide the search process. Hanafi et al. (2020) study a new variant of the TOP where precedence constraints are introduced. Each customer has a set of tasks that have to be accomplished according to a predefined order by an heterogeneous fleet of vehicles. If a customer is selected, then all the tasks have to be completed by possibly different vehicles. To tackle the problem, the authors proposed an enhancement of the Kernel Search (KS) framework that makes use of different sorting strategies and compare its performance to a Branch-and-Cut algorithm embedding the dynamic separations of different valid inequalities and the use of a simplified KS as primal heuristic.

To the best of our knowledge, only two applications of the TOP framework to the healthcare logistics are available in the literature. Jin and Thomas (2019) formulates he phlebotomist intrahospital routing problem as a TOP with stochastic rewards and service times. They present an a priori solution approach and derive a method for efficiently sampling the value of a solution, a value that cannot be determined analytically. The aim of the study in Aringhieri et al. (2022) is to find the best ambulance tours to transport the patients during a disaster in relief operations while considering fairness and equity to deliver services to patients in balance. The problem is formulated as a new variant of the TOP with hierarchical objectives to address also the efficiency issue.

Our work contributes to this variant of the TOP proposing two new hybrid algorithms based on machine learning and a neighbourhood search. Furthermore, it contributes to the application of the TOP framework to the healthcare logistics sector.

## Problem statement and mathematical formulation

We recall that the DSTC consists in organising the daily collection of swab tests reaching the house of the contact(s) of a positive case detected the day(s) before. A set of teams are in charge of collecting the swab tests along a path in the city.

We assume that (i) the number of required tests is larger than the daily capacity of all teams in terms of working time, and (ii) all tests to be collected are known in advance. We would remark that these assumptions represent the situation in which the pandemic is rapidly spreading over a given geographic area. Each team travels around the city collecting the swab tests. A priority is associated to each person in accordance with her/his health status and social connections. The priority represents the need of testing some people before other since they could become a spreader of the virus and/or they belong to more risky class of people (e.g., elderly and/or frail people). Such a priority drives the selection of which tests should be collected. Finally, time is crucial since we have to take into account both travel times and service times for collecting the swab(s) at home.

Let $P = \{ 1 , \ldots n \}$ be a set of places where a number $b_p$ ($p \in P$) of swab tests should be collected. The collection of the swab tests follows an integer priority $r_p$: the greater the priority is, the greater the importance of collecting such a swab test is. When the number of required tests are larger than the daily capacity, the priority represents the need of testing some people before other since they could become a spreader of the virus and/or they belong to more risky class of people (e.g., elderly and/or frail people). In our operational context, the priority is provided by the health authority since the DCT data are private. Further remarks are discussed in the conclusions.

For each place $p \in P$, we assume to have an estimate of the time $t^+_p$ and $t^-_p$ respectively needed to dress and to undress the *personal protective equipment*, the time $t^h_p$ to reach the house, and the time $t^s_p$ to collect a single swab test. Therefore, the overall time $t_p$ needed to perform all the operations needed to collect a swab test at the place $p \in P$ is equal to $t_p = t^+_p + 2 \, t^h_p + (b_p \, t^s_p) + t^-_p$. It is worth noting that $t^+_p$ and $t^-_p$ can be a constant but our choice is to be as general as possible in this description.

Let $M = \{ 1 , \ldots k \}$ be a set of medical teams in charge of collecting swab tests during their work-shift whose maximum duration is equal to $t_{\max }$. The teams start their work-shift from a *depot* 0 ending at the laboratory $n+1$. Depot and laboratory could be the same place. Considering $P^+ = P \cup \{ 0, n+1 \}$, let $t_{pq}$ be the travelling time to reach $q \in P^+$ from $p \in P^+$.

We are now ready to propose the integer linear programming model for the DSTC. Let us introduce the following decision variables:

- $x_{pqm} = 1$ if the team $m \in M$ visits the place $q \in P^+$ immediately after visiting the place $p \in P^+$, 0 otherwise;
- $y_{pm} = 1$ if the place $p \in P^+$ is visited by team $m \in M$, 0 otherwise;
- $u_{pm}$ is an integer representing the position of the place $p \in P^+$ in the path of the team $m \in M$.

The objective function seeks to maximise the overall priority of the swab tests collected:

1 $$\begin{aligned} \max z = \sum _{m \in M} \sum _{p \in P} r_p \, b_p \, y_{pm} . \end{aligned}$$

The rationale behind the idea of multiplying the priority for the number of requests on a place is that the collection of a greater number of swab tests is to be preferred. This is under the assumption that all the requests on a place are satisfied when it is visited, and is coherent with the objective of maximising the sum of priority scores associated with the collection of single swab tests. For instance, suppose to have among all the requests: (i) a place $p_0$ with priority score *r* and $b > 1$ tests to be collected, and (ii) *b* place $p_1, \ldots , p_b$ with the same score *r* and only one test to be collected in each one. Since the objective is to maximise the sum of the scores, visiting $p_0$ gives the same overall score *br* of visiting all $p_1, \ldots , p_b$, as we would like. On the other hand, the value of $r_p$ depends on the instance. If we would specify a different score $r_{pi}$ for each patient of the place *p*, then we can fix $r_p = \frac{\sum _i r_{pi}}{b_p}$, that is equivalent under the assumption of satisfying all the requests on a place when it is visited. Finally, it is worth noting that this objective function recalls the Clustered Team Orienteering Problem (Yahiaoui et al., 2019).

Constraint 2 ensures that each team starts its work-shift from the depot ending at the laboratory:

2 $$\begin{aligned} \sum _{m \in M} \sum _{q \in P} x_{0qm} = \sum _{m \in M} \sum _{p \in P} x_{p(n+1)m} = k , \end{aligned}$$

where *k* is the number of teams. Constraints 3 ensure that every place is visited at most once:

3 $$\begin{aligned} \sum _{m \in M} y_{pm} \le 1 , \qquad p \in P . \end{aligned}$$

Constraints 4 guarantee the connectivity of the work-shift of each medical team:

4 $$\begin{aligned} \sum _{q \in P \cup \{ 0 \}} x_{qpm} = \sum _{q \in P \cup \{ n+1 \}} x_{pqm} = y_{pm} , \qquad p \in P , m \in M . \end{aligned}$$

Constraints 5 ensure that the duration of each work-shift is less than or equal to the maximum duration:

5 $$\begin{aligned} \sum _{p \in P \cup \{ 0 \}} \sum _{q \in P \cup \{ n+1 \}} t_{pq} x_{pqm} + \sum _{p \in P} t_p y_{pm} \le t_{\max } , \qquad m \in M . \end{aligned}$$

Finally, the constraints 6 and 7 are necessary to prevent subtours in accordance with the Miller-Tucker-Zemlin formulation for the Travelling Salesman Problem (TSP) (Miller et al., 1960):

6 $$\begin{aligned}{} & {} 2 \le u_{pm} \le n+2 , \qquad p \in P \cup \{ n+1 \} , m \in M . \end{aligned}$$

7 $$\begin{aligned}{} & {} u_{pm} - u_{qm} + 1 \le ( n + 1 ) (1 - x_{pqm}) , \qquad p,q \in P \cup \{ n+1 \} , m \in M . \end{aligned}$$

## Ad hoc solution algorithms

As reported in Sect. 3, the DSTC is formulated as a variant of the TOP, which has been proved to be NP-hard (Butt and Cavalier, 1994). As soon as the complexity of the instance increases due to an increase of the number of places and/or the number of teams, an *ad hoc* and more efficient algorithm for solving the DSTC problem is required. In this section we report two new solution algorithms sharing the idea of computing the initial solution exploiting the clustering approach described in Sect. 4.1. For both algorithms (Sects. 4.2 and 4.3), first we provide a general description to introduce the main elements of the algorithm. Then, a detailed description of such elements is provided. It is worth noting that our algorithms share several ideas with the Tabu Search methodology (Glover and Laguna, 1997).

### Initial solution based on a Machine Learning approach

The two algorithms share the same procedure to compute the initial solution, which is inspired by a Machine Learning approach to clustering. As a matter of fact, the initial set *P* can be partitioned in *k* clusters, one for each team $m \in M$. In our settings we adopt two well known algorithms: k-means (MacQueen, 1967) and spectral clustering (Ng et al., 2001).

The k-means clustering algorithm determines a partition of a set of observations into a given number of clusters in order to minimise the sum of the square distances of the observations from the centre of their closest cluster, that is the one to which they belong. The spectral clustering is an algorithm used to identify communities of nodes in a graph based on the edges connecting them. It makes use of a similarity matrix that consists in a quantitative assessment of the relative similarity of each pair of nodes in the graph or points in the dataset.

The procedure initialSolution(*P*) returns a partition $[P^1 , \ldots P^k]$ and a solution *S* composed of $T^1 , \ldots , T^k$ tours: first, a partition $[P^1 , \ldots , P^k]$ of *P* is computed exploiting one of the above clustering algorithm, and then such a partition is the input for a procedure that computes a tour $T^m$ for each cluster $P^m$. Each tour starts from 0, traverses a subset of places belonging to the cluster $P^m$, and ends in $n+1$, and is computed as follows.

First, a $0-1$ knapsack problem (Martello and Toth, 1990) is solved, in which the places belonging to the cluster $P^m$ are the elements to be inserted in the knapsack whose capacity is set to $t_{\max }$, and each element/place $p \in P^m$ has weight equals to the service time $t_p$ and profit equals to $r_b \, b_p$. This instance of the $0-1$ knapsack is solved by applying the classic dynamic programming algorithm (Martello et al., 1999). The subset of places selected by solving the $0-1$ knapsack are then the input for a TSP procedure to compute the tour $T^m$. In our implementation, such a tour is computed using a TSP heuristic starting from the depot 0 and modifying the solution obtained by connecting the last node to the laboratory instead of coming back to the depot. We tested both the Christofides (Christofides, 1976) and the Lin-Kernighan (Lin and Kernighan, 1973) algorithms.

### Clustering search CS1 algorithm


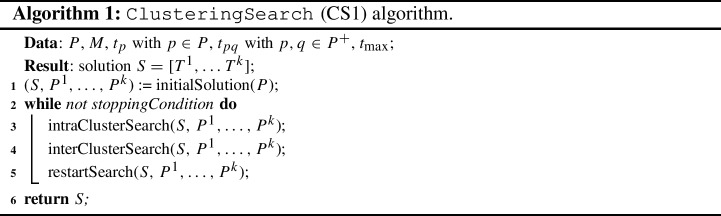


As depicted in Algorithm 1, the algorithm ClusteringSearch CS1 starts computing an initial solution using the procedure initialSolution(*P*). This initial solution is then improved during a cycle repeated until the stopping condition is met, that is the algorithms terminates after a number $NI_1$ of not improving iterations. At each iteration, the improvement phase consists in two steps. The intraClusterSearch tries to improve the tour of each cluster exchanging places in the tour with places not yet visited but in the same cluster. On the contrary, the interClusterSearch tries to improve the tour of a cluster by moving places from a different cluster. Finally, after a number of not improving iterations, a restart is performed to diversify the search.

*Procedure* intraClusterSearch. In order to improve the quality of each single tour $T^m \subseteq P^m$, we implemented a straightforward neighbourhood search based on a simple swap between a pair of places $p_1 \in T^m$ and $p_2 \in P^m \setminus T^m$. Such a neighbourhood is completely explored to determine the best feasible move (*best improvement*), that is the pair of places returning the tour having the highest value given by the sum of the values $r_b \, b_p$ for each $p \in T^m$. The neighbourhood search can accept also not improving moves, that is moves determining tours whose values is less than or equal to the incumbent one. To avoid cycles among already visited solutions, two tabu lists $L_1$ and $L_2$ - based on tabu tags (Gendreau et al., 1994) - has been implemented in such a way to avoid a place to be moved from or into a tour for the next $\ell _1$ and $\ell _2$ moves, respectively. Such a neighbourhood search is repeated 100 times.

*Procedure* interClusterSearch. Since the quality of the tour $T^m$ can depend on the initial clustering, we developed an improvement phase based on the idea of moving places from a cluster to another one. Basically the procedure interClusterSearch tries to improve the quality of the tour $T^m$ by moving a place $p \in P^{v}$ in the cluster $P^{m}$, with $v \in M$ and $v \ne m$.

Such a neighbourhood is completely explored to determine the best feasible move (*best improvement*). The neighbourhood search can accept also not improving moves. To avoid cycles among already visited solutions, a tabu list $L_3$ - based on tabu tags (Gendreau et al., 1994) - has been implemented in such a way to avoid a place to be moved from the destination cluster for the next $\ell _3$ moves. Each single move is evaluated by computing the knapsack problem as in the procedure initialSolution on the tentative cluster $P^{m} \cup \{ p \}$.

After selecting the best move, that is the pair $(p, P^{m})$ maximising the knapsack solution, the tour $T^{m}$ is re-computed as in the procedure initialSolution. If the resulting tour $T^m$ improves the previous one, a simple intensification is performed by repeating the procedure intraClusterSearch.

Such a neighbourhood is also used to have a weak diversification of the search: actually, after a number of neighbourhood explorations $W_1$, we force to move a place $p \in P^{v}$ such that it belongs to the tour $T^v$.

The procedure interClusterSearch is repeated until a maximum number of not improving iterations $I_1$ is reached.

*Procedure* restartSearch. After the end of the interClusterSearch procedure, a restart method is applied in order to diversify the search. For each cluster, the restart randomly moves the $25\%$ of the places to the closest one. After that, the tours for the modified clusters are re-computed in accordance with the same procedure used in initialSolution(*P*).

### Clustering search CS2 algorithm

As depicted in Algorithm 2, the algorithm ClusteringSearch CS2 starts computing an initial solution using the procedure initialSolution(*P*). This initial solution is then improved during a cycle repeated until the stopping condition is met, that is the algorithms terminates after reaching a maximum number $MI_2$ of iterations. At each iteration, the improvement phase consists in two steps. The increaseTour tries to improve the current tour by adding a place not belonging in any tours in such a way to do not exceed its maximum duration. The modifyTour tries to improve the current tour by swapping a place belonging to a tour with a place not belonging in any tours. Finally, after a number of not improving iterations, a restart is performed to diversify the search.
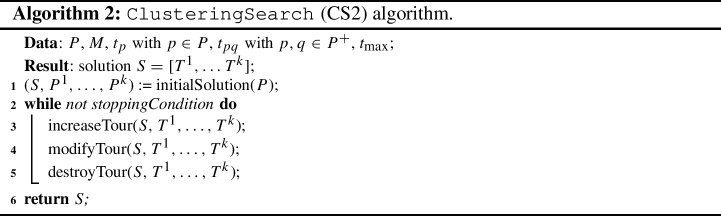


*Procedure* increaseTour. The increaseTour implements a simple neighbourhood search whose target is to improve the contribution of the $T^m$ to the objective function without exceeding the maximum duration $t_{\max }$.

Let $P^{out}$ be the set of the places that do not belong to any tours, that is $P^{out} = P \setminus \{ T^1 \cup \ldots \cup T^k \}$. For each tour $T^1 \cup \ldots \cup T^k$, the search consists in evaluating the insertion of a place $p \in P^{out}$ in the tour $T^m$ in a greedy way: for each pair of places $q_1$ and $q_2 \in T^m$, we try to insert the place *p* between $q_1$ and $q_2$ by considering the new possible tour $T^m$ given by the sequence $[\ldots ,q_1,p,q_2,\ldots ]$ instead of $[\ldots ,q_1,q_2,\ldots ]$; if the resulting tour is feasible, it will be considered as the best move at the end of the neighbourhood exploration. Note that in the case of two moves determining a tour with the same values, the search will favour that solution with minimal tour duration.

Such a neighbourhood is completely explored to determine the best feasible move (*best improvement*). At the end of the exploration, the best move is applied modifying a tour and increasing the whole value of objective function determining a new best solution *S*. The increaseTour ends as soon as a new place insertion is not found.

*Procedure* modifyTour. After the increaseTour, we expect that the only way to improve a tour $T^m$ is a swap between a place $p \in P^{out}$ and a place $q \in T^m$. The modifyTour implements a neighbourhood search based on this simple idea.

For each place $p \in P^{out}$ and for each tour $T^m$, the search consists in evaluating the swap between the pair (*p*, *q*) with $q \in T^m$ by considering the new possible tour $T^m$ given by the sequence $[\ldots ,q_1,p,q_2,\ldots ]$ instead of $[\ldots ,q_1,q,q_2,\ldots ]$; if the resulting tour is feasible, it will be considered as the best move at the end of the neighbourhood exploration. Again, the neighbourhood is fully explored to determine the best feasible move (*best improvement*). Note that in the case of two moves determining a tour with the same values, the search will favour that solution with minimal tour duration.

The neighbourhood search can accept also not improving moves, that is moves determining tours whose values is less than or equal to the incumbent one. To avoid cycles among already visited solutions, we adopt the same tabu list $L_1$ and $L_2$ already introduced for intraClusterSearch but with different values of $\ell _1$ and $\ell _2$ moves, respectively.

The procedure modifyTour is repeated until a maximum number of not improving iterations $I_2$ is reached.

*Procedure* destroyTour. After the end of the modifyTour procedure, a restart method is applied in order to diversify the search. For each tour, the restart deletes a third of the places $p \in T^m$. Such places are those having the lower ratio between the overall tour priority and the tour duration. The diversification is guaranteed by the fact that such places are inserted in the tabu list $L_2$, which avoids a place to be re-inserted in its origin tour for the next $\ell _2$ moves. By consequence, such places are not considered during the next increaseTour application.

## Quantitative analysis

In this section we report a quantitative analysis performed on a set of realistic instances (depicted in Sect. 5.1) in order to evaluate (i) the possibility of solving the DSTC problem in a realistic operational context, and (ii) the quality and the efficiency of the two algorithms proposed for its solution (reported in Sect. 5.2 and Sect. 5.3).

### Description of the instances

A set of 54 instances has been randomly generated in order to test the impact of the optimisation on realistic scenarios based on the city of Turin, Italy, which has a surface of $130 km^2$ and a population of 887, 000 inhabitants. Each instance is defined starting from (i) a set of nodes (representing the locations of the depot, the laboratory and the places), (ii) a probability distribution that assigns an integer score in [1, 100] to the places, and (iii) a fixed number of teams *k*.

Five different set of nodes $N_1,\ldots ,N_5$ are taken from the test instances for the TOP provided in (Tsiligirides, 1984; Chao et al., 1996a), which are downloadable on the KU Leuven website (www.mech.kuleuven.be/en/cib/op). Since such sets have cardinality between 21 and 102, we generated with a uniform distribution two further sets of nodes $N_6$ and $N_7$, with cardinality 150 and 200 respectively, in order to have medium size instances. Finally, two further sets of nodes $N_8$ and $N_9$, with cardinality 907 and 2149 respectively, have been generated in order to have bigger and more challenging instances. The number of nodes of instances $N_8$ and $N_9$ have been computed taking into account the actual daily swab tests collected at the beginning of the second wave of the Covid-19 pandemic, when the DCT was available in Italy. We considered the average ($N_8$) and the maximum ($N_9$) number of regional swab tests in the period between 22nd September and 21st October, 2021 (lab24.ilsole24ore.com/coronavirus/) multiplied for 0.2, that is approximately the fraction of the inhabitants of the city of Turin on the total of Piedmont region population. Finally, we fixed the number of nodes in such a way to have an expected value of the required swab tests equal to the half of the estimate of the swab tests executed in the city of Turin, that is the scenario in which the 50% of them need to be collected at home.

**Fig. 2:**
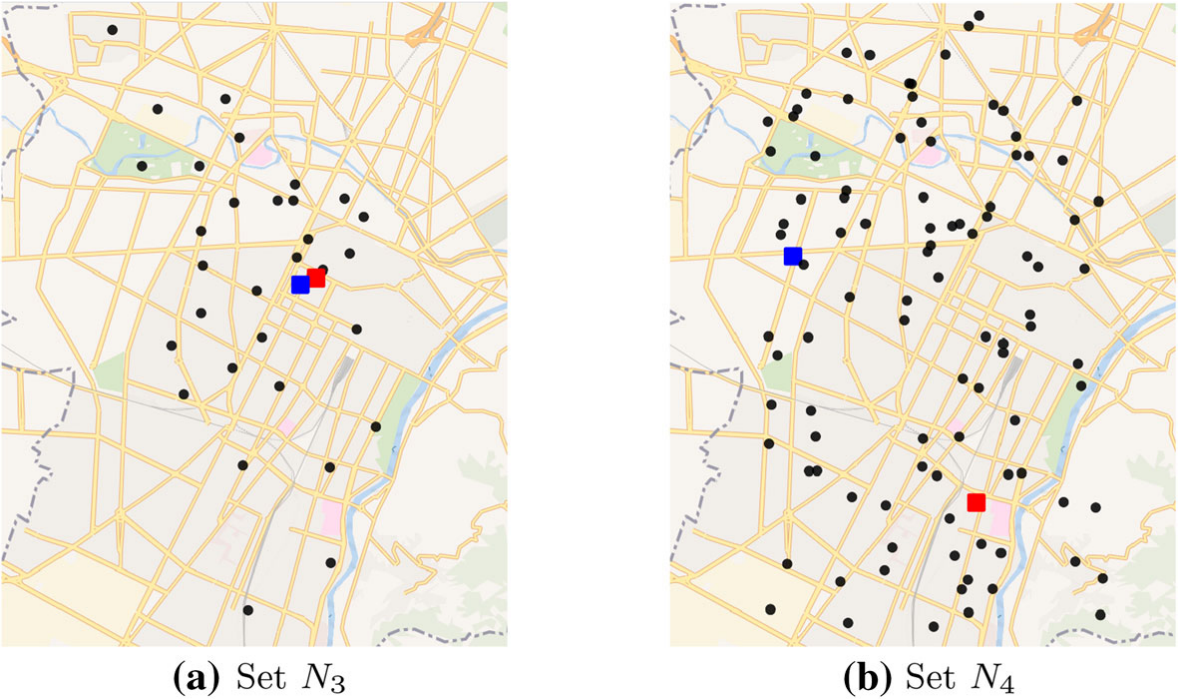
Graphical representation of the set of nodes on the city of Turin

**Fig. 3:**
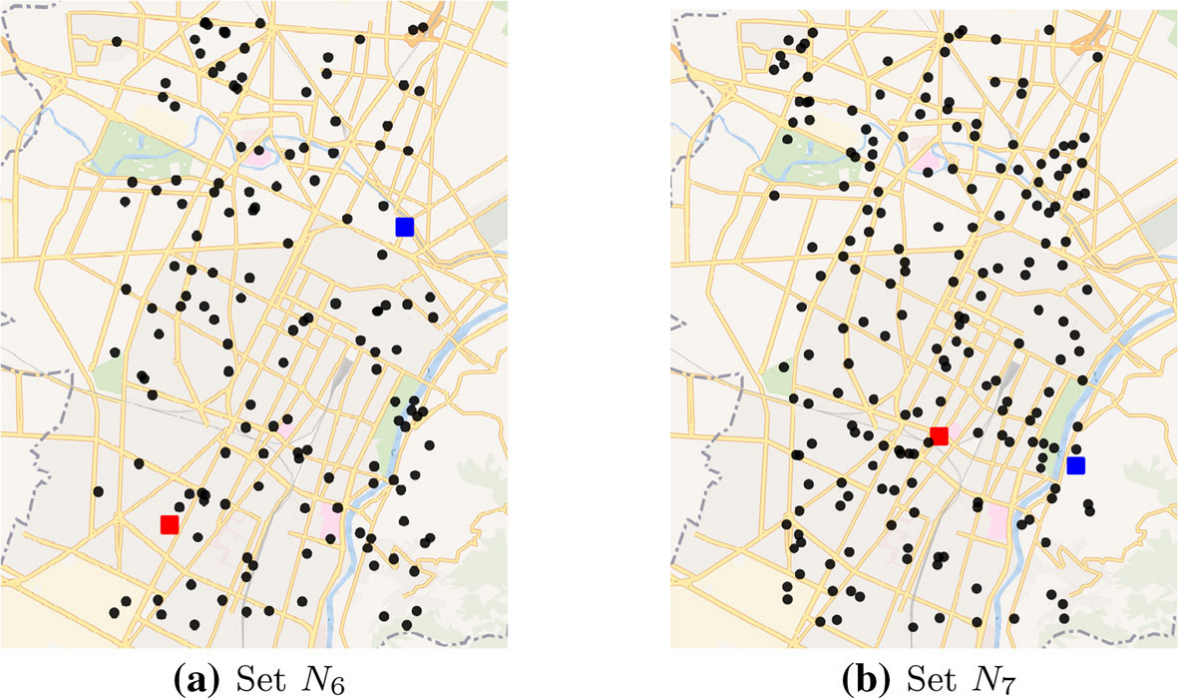
Graphical representation of the set of nodes on the city of Turin

All set of nodes are scaled on the area of Turin as depicted in Figs. 2 and 3 in which the red and the blue squares represent the depot and the laboratory, respectively.

Travelling distances are derived computing the 1-norm between each pair of nodes and considering an average speed of 20km/h. We decided to compute distances with the 1-norm because of the “checker-board structure” of most of the city, resulting in a better approximation than the classic Euclidean distance.

Furthermore, for each node representing a place $p \in P$, we generate the number of swab $b_p$ to be executed using the distribution of the households in accordance with the data of the Statistic Office of the City of Turin (http://www.comune.torino.it/statistica/): the families with 1, 2, 3, 4, 5, and 6+ members are respectively the 46.99%, 26.84%, 14.12%, 9.10%, 2.13%, and 0.82%. The rationale of this choice is that each place can correspond to a family, and if a person needs a test, then all the family members could have a contact with her/him.

For each set of nodes representing a place $p \in P$, we generate the priority $r_p$ of visiting a node using two different distributions in [1, 100]: a discretised uniform and a discretised cumulative exponential.

The maximum duration $t_{\max }$ and the number of the teams/tours *k* have been set in such a way to guarantee that the available resources are not sufficient to collect all the swabs, fixing $t^s_p = 3$ minutes for each swab test plus $t^+_p + 2 \, t^h_p + t^-_p = 5$ minutes for the additional time spent on the place $p \in P$.

For each set of nodes, we used the 1-tree bound (Valenzuela and Jones, 1997) for the TSP as lower-bound of the travelling time needed to visit all the nodes with only 1 team. We sum the whole service time $t_p$ to the 1-tree bound value in order to have a lower bound *L* of the total time. Then, we fixed $t_{\max }$ in such a way that it results as a multiple of 30 minutes less than or equal to 9 hours, and $k \, t_{\max } < L$. The characteristics of the 54 instances are reported in Table 1 in which the letters U and E stand for uniform and exponential distributions used to generate the priority.

**Table 1:** Summary of the 54 instances used in our quantitative analysis

Starting set	$|P^+|$	$t_{\max }$	*k*	**id**	Starting set	$|P^+|$	$t_{\max }$	*k*	**id**
$N_1$	32	150	1	N1U1	$N_5$	102	420	1	N5U1
			2	N1U2				2	N5U2
			3	N1U3				3	N5U3
			1	N1E1				1	N5E1
			2	N1E2				2	N5E2
			3	N1E3				3	N5E3
$N_2$	21	90	1	N2U1	$N_6$	150	540	1	N6U1
			2	N2U2				2	N6U2
			3	N2U3				3	N6U3
			1	N2E1				1	N6E1
			2	N2E2				2	N6E2
			3	N2E3				3	N6E3
$N_3$	33	120	1	N3U1	$N_7$	200	540	2	N7U2
			2	N3U2				3	N7U3
			3	N3U3				4	N7U4
			1	N3E1				2	N7E2
			2	N3E2				3	N7E3
			3	N3E3				4	N7E4
$N_4$	100	360	1	N4U1	$N_4$	100	360	1	N4E1
			2	N4U2				2	N4E2
			3	N4U3				3	N4E3
$N_8$	907	540	10	N8U1	$N_9$	2149	540	23	N9U1
			15	N8U2				35	N9U2
			20	N8U3				47	N9U3
			10	N8E1				23	N9E1
			15	N8E2				35	N9E2
			20	N8E3				47	N9E3

### Computational analysis

The aim of this section is to report the quantitative analysis on the realistic instances described in the previous section. First we summarise the analysis performed using a general purpose solver to solve the DSTC problem. Then, we discuss the efficiency and the quality of the solutions computed by our proposed algorithms. The computational tests have been performed on a standard desktop computer equipped with a Intel Core i7-8700 3.20GHz with 12 cores, and 16 Gb of memory.

The integer linear program (1)–(7) has been implemented in Python adopting the Pyomo optimisation library (Hart et al., 2017) and CPLEX 12.9 as general purpose solver. The main settings of CPLEX are the default ones except for the relative MIP gap tolerance, which has been set to $3\%$ and $1\%$ respectively in two subsequent tests. The rationale is to evaluate the running time required by a general purpose solver to compute a good quality solution for the DSTC. Then, we compare these results with the solutions computed by our algorithms. In other words, we will use CPLEX as competitors of our algorithms.

**Table 2:** Summary of the results obtained with CPLEX as general purpose solver

	cplex 3%		cplex 1%			cplex 3%		cplex 1%	
id	Best	secs	Best	secs	id	Best	secs	Best	secs
N1U1	1301	0.2	1311	0.2	N4E1	3850	11.5	3850	10.2
N1U2	2207	52.0	2207	992.6	N4E2	6676	1998.9	6676	1826.4
N1U3	2782	29.1	2784	3754.8	N4E3	8755	1187.7	8747	1019.1
N1E1	1772	0.2	1772	0.2	N5U1	3793	14.1	3853	39.0
N1E2	3344	440.8	3345	5207.0	N5U2	6103	1498.3	6181	3671.9
N1E3	3668	2699.4	3668	3836.3	N5U3	7459	1185.7	7425	1176.3
N2U1	675	0.0	675	0.0	N5E1	4273	1.5	4309	9.5
N2U2	1123	0.2	1123	2.2	N5E2	7388	403.8	7445	2997.7
N2U3	1465	144.4	1465	52.5	N5E3	9198	1158.2	9273	1026.7
N2E1	837	0.0	837	0.0	N6U1	5201	33.6	5264	36.0
N2E2	1270	0.5	1270	0.5	N6U2	8632	698.8	8651	1647.6
N2E3	1680	3119.4	1680	3051.7	N6U3	10230	1920.0	10689	2093.2
N3U1	850	0.1	850	0.0	N6E1	3806	34.1	3821	287.2
N3U2	1921	0.8	1921	4.0	N6E2	6278	2650.3	6465	8281.3
N3U3	2360	4654.9	2360	4625.6	N6E3	7874	1812.2	8276	7148.1
N3E1	1124	0.2	1124	0.1	N7U2	10831	8023.3	10937	5587.6
N3E2	1513	4.8	1513	12.5	N7U3	12653	2244.5	12653	2239.5
N3E3	2586	294.8	2586	5006.3	N7U4	14408	3613.3	14135	3317.9
N4U1	3714	5.7	3714	23.8	N7E2	6834	1381.8	6801	1383.4
N4U2	5955	729.9	6046	469.5	N7E3	8810	2093.0	7866	1908.5
N4U3	7623	822.7	7644	2717.9	N7E4	10359	9867.8	10152	8225.6
	Avg. time Cplex $3\%$: 1305.5 secs					Avg. time Cplex $1\%$: 1992.6 secs			

Table 2 reports the computational results of the general purpose solver with different relative MIP gap tolerance on the first 42 instances belonging to the sets $N_1$–$N_7$: the columns “best” and “secs” report the value of the best integer solution computed and the running time required in seconds, respectively. Such results proved the increasing complexity of the problem as soon as the number of teams *k* and/or the number of places *n* increases. For this reason, we do not consider the instance with $k=1$ in the next comparisons since they can be easily solved. We would remark that the general purpose solver stopped its computation (for an out of memory error) before reaching the requested gap for an unexpected number of the medium size instances. This number increases as soon as the MIP gap is set to $1\%$. For the sake of completeness, we report an average running time of 621.6 and 825.8 for the same test reported in Table 2 but with MIP gap set to $10\%$ and $5\%$, respectively.

Table 3 reports the comparisons between our algorithms and the general purpose solver on the instances belonging to the sets $N_1$–$N_7$: the columns “best”, “secs” report respectively the values of the best solution and the running time in seconds for each considered algorithm; the columns “gap cplex 3%” and “gap cplex 1%” report the relative gap of the two proposed algorithms with respect to the solution computed by CPLEX with different relative MIP gap tolerance, respectively. The computational results of our algorithms are obtained with the following settings: $NI_1 = \{500,1000,2000\}$, $W_1 = \{5,8,15\}$ and $I_1 = \{15,25,50\}$, $MI_2 = \{50000,10000\}$ and $I_2 = n$; the lengths of the tabu lists are $\ell _1 = 6$, $\ell _2 = 10$, $\ell _3 = 10$ for CS1, and $\ell _1 = 5$, $\ell _2 = 12$ for CS2. Further, we used the spectral clustering in our computational tests. This parameter setting has been selected among others since it is the one providing better average results.

**Table 3:** Comparing the results of Clustering Search algorithms with CPLEX with a given MIP gap on the instances with $k>1$

id	init	CS1		CS2		Gap cplex 3%		Gap cplex 1%	
	Best	Best	secs	Best	secs	CS1 (%)	CS2 (%)	CS1 (%)	CS2 (%)
N1U2	2181	2181	49.6	2207	36.0	−1.2	0.0	−1.2	0.0
N1U3	2643	2709	38.4	2766	38.8	−2.6	−0.6	−2.7	−0.6
N1E2	3235	3344	58.3	3340	37.0	0.0	−0.1	0.0	−0.1
N1E3	3409	3525	52.6	3631	39.9	−3.9	−1.0	−3.9	−1.0
N2U2	951	1026	36.3	1123	15.6	−8.6	0.0	−8.6	0.0
N2U3	1175	1422	21.7	1464	20.4	−2.9	−0.1	−2.9	−0.1
N2E2	1071	1235	21.9	1270	16.1	−2.8	0.0	−2.8	0.0
N2E3	1481	1645	35.7	1680	19.4	−2.1	0.0	−2.1	0.0
N3U2	1681	1921	63.8	1921	36.9	0.0	0.0	0.0	0.0
N3U3	1995	2215	37.3	2360	44.7	−6.1	0.0	−6.1	0.0
N3E2	1234	1467	25.8	1513	33.8	−3.0	0.0	−3.0	0.0
N3E3	2336	2385	27.6	2514	44.7	−7.8	−2.8	−7.8	−2.8
N4U2	5571	5702	153.1	5939	139.3	−4.2	−0.3	−5.7	−1.8
N4U3	7258	7269	149.3	7417	150.2	−4.6	−2.7	−4.9	−3.0
N4E2	6301	6397	105.0	6660	134.9	−4.2	−0.2	−4.2	−0.2
N4E3	8382	8572	115.5	8591	153.0	−2.1	−1.9	−2.0	−1.8
N5U2	6140	6143	192.3	6140	145.2	0.7	0.6	−0.6	−0.7
N5U3	7684	7684	194.2	7684	143.2	3.0	3.0	3.5	3.5
N5E2	7074	7276	168.0	7309	145.8	−1.5	−1.1	−2.3	−1.8
N5E3	9518	9571	162.0	9592	147.4	4.1	4.3	3.2	3.4
N6U2	8348	8348	212.2	8434	224.5	−3.3	−2.3	−3.5	−2.5
N6U3	10939	10939	220.7	10939	225.3	6.9	6.9	2.3	2.3
N6E2	6132	6202	249.5	6264	213.4	−1.2	−0.2	−4.1	−3.1
N6E3	8135	8212	226.9	8238	208.8	4.3	4.6	−0.8	−0.5
N7U2	10695	10757	220.1	10821	347.6	−0.7	−0.1	−1.6	−1.1
N7U3	14057	14127	224.4	14217	345.2	11.6	12.4	11.6	12.4
N7U4	14041	15649	392.6	16581	396.8	8.6	15.1	10.7	17.3
N7E2	7094	7108	221.7	7239	312.1	4.0	5.9	4.5	6.4
N7E3	9469	9469	222.7	9476	342.0	7.5	7.6	20.4	20.5
N7E4	9481	10452	290.1	11028	353.2	0.9	6.5	3.0	8.6
Avg. values			**139.6**		**150.4**	**−0.4**	**1.8**	**−0.4**	**1.8**

The results reported in Table 3 prove the capability of our algorithms to compute good quality solutions saving a large amount of running time ranging between $88\%$ and $93\%$. The gaps with initialSolution(*P*) ranges between the $4.9\%$ and the $7.2\%$, which prove their capability to improve the initial solution by escaping from local optima.

In terms of pure solution quality (discarding the running time), algorithm CS1 is less competitive on average with respect to the general purpose solver: while the solver is better on smaller instances, algorithm CS1 performs better on the larger ones. On the contrary, algorithm CS2 computes on average better solution than the general purpose solver, especially on larger instances. Summing up, the gaps between our algorithms and the general purpose solver largely increases as soon as the complexity of the instances increases in terms of number of places and/or number of teams. For the sake of completeness, the comparison of our algorithms with the general purpose solver with MIP gap set to $10\%$ and $5\%$ showed that the two algorithms compute better solutions than the solver: about $2.8\%$ and $5.0\%$ (MIP gap set to $10\%$) and $0.7\%$ and $2.9\%$ (MIP gap set to $5\%$). Although not explicitly listed, we would like to remark that the algorithms CS1 and CS2 perform very well also in the instances having $k=1$.

From the proposed analysis emerges the fact that the instances whose scores are generated by a discretised uniform seems easier than those with a score generated by a discretised cumulative exponential. This fact can be explained considering that the former distribution generates scores that are more spread in the interval [1, 100] with respect to the latter, which concentrates the scores in the values closest to 100. Accordingly, solutions of instances with uniform scores are more heterogeneous than the ones with cumulative exponential scores, which could lead to symmetry issues.

Table 4 reports the computational on the larger instances belonging to the sets $N_8$ and $N_9$: the columns “best” and “secs” report respectively the values of the best solution and the running time in seconds for each considered algorithm; the columns “gap init” and “gap CS1” report the relative gap of the two proposed algorithms with respect to the solution computed by initialSolution(*P*) and the CS1 algorithm, respectively. Such computational results are obtained by changing only the parameters concerning the stopping conditions, that is $NI_1 = 100$ and $MI_2 = 1000$. We would remark that CPLEX results are not reported since it is not able to compute a good solution in a reasonable running time.

The results reported in Table 4 prove the better efficiency and quality of algorithm CS2 with respect to CS1: as a matter of fact, CS2 is capable to computer, on average, better solutions (about 20.41%) than CS1 in less running time (CS2 is about ten times faster than CS1). Furthermore, the bigger gaps with initialSolution(*P*) confirm their capability to improve the initial solution by escaping from local optima.

**Table 4:** Comparing the results of Clustering Search algorithms on the larger instances

id	init	CS1		CS2		CS1	CS2	
	Best	Best	secs	Best	secs	Gap init (%)	Gap init (%)	Gap CS1 (%)
N8E1	25937	58082	2378	65387	267	123.93	152.10	12.58
N8U1	21660	47156	3741	49650	268	117.71	129.22	5.29
N8E2	26202	74438	3024	81897	277	184.09	212.56	10.02
N8U2	22073	58988	7376	63598	329	167.24	188.13	7.82
N8E3	27777	83485	5457	97551	340	200.55	251.19	16.85
N8U3	22567	65272	10312	74572	338	189.24	230.45	14.25
N9E1	44785	129892	17285	148674	1549	190.03	231.97	14.46
N9U1	38188	108222	18981	116239	1576	183.39	204.39	7.41
N9E2	46899	142753	25912	189598	1628	204.38	304.27	32.82
N9U2	39581	105981	24841	149814	2060	167.76	278.50	41.36
N9E3	49968	156584	28459	222044	3095	213.37	344.37	41.81
N9U3	41286	125468	31587	175937	2273	203.90	326.14	40.22
Avg. values			**14946**		**1167**	**178.80**	**237.77**	**20.41**

**Table 5:** Evaluating the impact of solutions over different categories of patients

	% Visited by cardinality						% Tested by score					% Tested by dist.			
id	1	2	3	4	5	6	Min.	Low	Avg.	High	Max.	A	B	C	D
N9E1	1	33	48	56	68	63	0	0	20	36	67	38	29	40	31
N9U1	2	29	52	55	74	75	0	10	40	60	72	38	34	30	65
N9E2	4	59	66	67	81	88	2	12	38	50	55	52	47	52	46
N9U2	10	53	64	68	85	81	0	34	68	73	81	51	51	49	65
N9E3	36	60	66	68	81	88	3	26	62	71	91	60	56	59	50
N9U3	32	62	72	75	90	88	7	50	74	83	96	62	62	59	71
Avg.	**14**	**49**	**61**	**65**	**80**	**80**	**2**	**22**	**50**	**62**	**77**	**50**	**46**	**48**	**55**

### Fairness and managerial insights

Although we shown the effectiveness of the proposed approach in determining a near-optimal solution of the DSTC problem, several managerial issues could not be directly deducible from the results reported in Tables 3 and 4 . In fact, the modelling choice to use an objective function representing an overall social cost or the behaviour of the proposed algorithm could lead to undesirable effects from the point of fairness, with some categories of patients benefiting at the expense of others. For this reason, we present a more detailed analysis of the solutions determined by the CS2 algorithm, where we consider the fraction of patients who are selected on the basis of the urgency to be tested, the number of family members, and the location of their home in the geographical area on which the swab tests need to be collected.

In Table 5 we report the percentage of tested patients in the solution provided by the algorithm CS2, dividing the nodes of the graph with respect to three different characteristics: the cardinality, the score, and the sum of the distance from the depot and from the laboratory. The scores have been divided into 5 classes: *min* (1–20), *low* (21–40), *avg* (41–60), *hig* (61–80), and *max* (81–100). Furthermore, the geographic area has been divided into 4 sub-areas, depending by the ratio between the sum of the distance between the node and both the depot and the laboratory, and the distance between the depot and the laboratory: *A* (1.0 – 1.4), *B* (1.4 – 1.8), *C* (1.8 – 2.2), and *D* (2.2 – 2.6). From a geometrical point of view, the borders between the 4 sub-areas could be seen as 4 concentric ellipses having the depot and the laboratory as foci. We remark that these all nodes’ attributes has been considered as independent during the instance generation, and we present results about the larger instances, since they are the more significant from a statistical point of view.

**Fig. 4:**
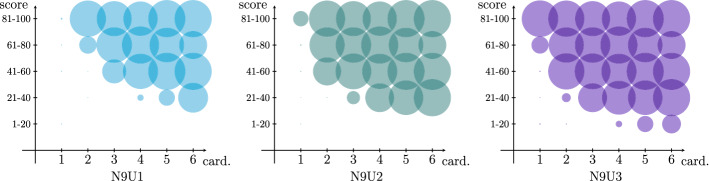
Percentage (bubble dimension) of visited nodes by cardinality (x-axis) and score (y-axis) for instances with uniformly distributed scores and different number of teams

As expected, the cardinality and the score are very relevant on the fraction of the nodes selected in the solution. While this is desired with regard to scores, small families are disadvantaged, especially when the resources available are scarce. Indeed, less than 10% of nodes corresponding to mononuclear families are visited when the number of teams is equal to 23 (instances N9E2 and N9U2) or 35 (instances N9E2 and N9U2). This means that most of nodes with higher score and cardinality 1 and 2 are not visited because the time needed for their test collection would decrease the overall social cost. This phenomenon can be better seen in Fig. 4, where 30 different groups of nodes are considered on the basis of both the cardinality and the score class. The impact of increasing the number of teams can be further observed by comparing the three bubble charts, where the coverage is guaranteed also for the more urgent mononuclear families as soon as the quantity of resource increases. Finally, we observe that scores generated with exponential distribution amplify this effect. Therefore, the issue of fairness between families with different number of components should be discussed with the decision maker when defining the score to be associated to each node, since it could lead to different trade-offs between the number of traced infected patients and level of fairness. From a managerial point of view, a what-if analysis by comparing the characteristics of solution provided by different scores using our approach is suggested.

**Fig. 5:**
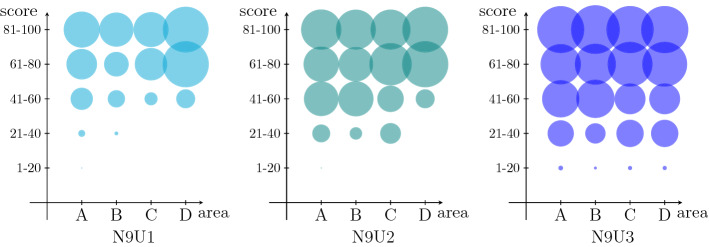
Percentage (bubble dimension) of tested patients by geographical area (x-axis) and score (y-axis) for intances with uniformly distributed scores and different number of teams

On the contrary, a greater distance from the depot and the laboratory does not seem to represent a disadvantage in terms of probability of being tested. As can be observed in the last four columns of Table 5, on average, the fraction of patients tested does not depend on how far they are from the start and end position of the tours. Sub-area *D* has counter-intuitively a slightly higher percentage of patients tested than the more central ones. This is also evident from Fig. 5, where fraction of tested patients divided by sub-area and score class is shown. We notice that the impact of the score is definitely superior with respect to the sub-area in which the patients are located. In particular, more urgent swabs in sub-area D are slightly more likely to be executed with respect other sub-areas, while the opposite happens for the lower score classes. Nevertheless, this phenomenon is attenuated by increasing the number of teams (instance N9U3). Finally, we remark that although the number of collected swab tests increases when more resources are available, a node can be visited in the solution with a certain number of teams and not visited in the solution with a higher one (the reverse is obvious). This fact has been observed several times in the solutions provided by the algorithm CS2 and further proves the need of a tool such as that proposed in this work to support complex decisions for the DSTC by computing non-trivial solutions.

## Conclusions

One of the main reasons of the limited spread of the DCT app in Italy was the fear of being trapped at home without being able to take the test urgently. This happened (and it was reported by many newspapers) in several northern cities when the pandemic was soaring. This lead us to investigate the DSTC problem as stated in the research question reported in the introduction.

We introduced a new optimisation problem arising in the daily management of a contact tracing system. We provided a mathematical formulation of the problem and two new algorithms for its solution. Our quantitative analysis considered a set of 54 instances randomly generated in order to test the impact of the optimisation on realistic scenarios based on the city of Turin, Italy. The quantitative analysis proved the possibility of solving the DSTC problem in a realistic operational context while the comparison with a general purpose solver proves the effectiveness and the efficiency of the proposed algorithms. Furthermore, it is worth noting that the computed solutions are not trivial: actually, some places with higher priority are “sacrificed” to visit a greater number of places with lower priority, in the name of a better result for the community. Summing up, we can answer positively to the our research question since we proved the capability (especially that of the algorithm CS2) to deal with real instances, that is those belonging to the benchmark sets $N_8$ and $N_9$. Further, from the specific analysis, it was ascertained that the solutions provided by the proposed approach do not present any critical issues in terms of fairness with respect to the geographical area to which the patients belong.

The DSTC poses a managerial implication that should be addressed by healthcare managers, that is how to deal with places that are not served during a day. There are several possibilities. One is to avoid such a situation with a proper forecasting of the the number of swab tests to be collected the day after (as reported in Aringhieri et al. (2017) for ambulance management) in order to determine the appropriate number of teams. Alternatively, the healthcare managers can decide to improve their priority in order to push them to the top of the list. We would recall that the choice of the priority scores $r_p$ is left to the decision maker, who can adopt a range of policies from maximising the absolute number of swab tests ($r_p = 1$ for all *p*) up to establish a hierarchical priority among the places (e.g., $r_p \gg r_p^{\prime }$ implies that the place $p^{\prime }$ can be visited only if *p* is also visited).

One of the possible extensions of this work is therefore the development of a prioritisation method. To this end, we can drawn inspiration from the work of Charkhgard et al. (2018) in which the authors studied the problem of minimising the spread of influenza virus infections in (dynamic) networks of people by isolating sick nodes (or vaccinating susceptible nodes) over time. From an optimisation perspective, this can be viewed as a problem of removing nodes with certain characteristics from networks of people over time. This problem belongs to the larger class of problems called *critical node problem* for which we developed several efficient optimisation algorithms (Aringhieri et al., 2016b; Addis et al., 2016; Aringhieri et al., 2016a). The challenge posed by the development of such a prioritisation method is to evaluate which nodes are critical, that is maximising the fragmentation of the resulting network. From this point of view, the distributed approach of many DCT apps makes this evaluation more difficult. The distributed approach is a way to foster privacy protections in the form of local storage of data on smartphones, which can be uploaded to the central system only after the approval of the person involved. The main effect is a limited knowledge of the dynamic network of possible infected people making more challenging the understanding which are the most critical nodes, that is those corresponding to people that should be tested before others.

Another possible extension of this work is to consider the possible dynamics of the DSTC problem operational context. We considered a static version of the problem in which all the swab tests are known in advance. Our work can be extended to include the case in which new swab tests to be collected might arrive over time. A possible solution is to develop online re-optimisation algorithms (Aringhieri, 2020).

## Footnotes

1 Url: https://ec.europa.eu/commission/presscorner/detail/en/ip_20_1904.
